# Simulation and Experimental Investigation on Carbonized Tracking Failure of EPDM/BN-Based Electrical Insulation

**DOI:** 10.3390/polym12030582

**Published:** 2020-03-05

**Authors:** Muhammad Tariq Nazir, Faizan Tahir Butt, Bao Toan Phung, Guan Heng Yeoh, Ghulam Yasin, Shakeel Akram, Muhammad Shoaib Bhutta, Shahid Hussain, Tuan Anh Nguyen

**Affiliations:** 1 School of Mechanical and Manufacturing Engineering, University of New South Wales Sydney, NSW 2052, Australia; g.yeoh@unsw.edu.au; 2 School of Electrical Engineering and Telecommunications, University of New South Wales Sydney, NSW 2052, Australia; engrfaiz87@gmail.com (F.T.B.); toan.phung@unsw.edu.au (B.T.P.); 3 State Key Laboratory of Chemical Resource Engineering, College of Energy, Beijing University of Chemical Technology, Beijing 100029, China; 4 Institut d’Electronique et des Systèmes (IES), UMR 5214, CNRS, Université de Montpellier, 34095 Montpellier, France; shakeel.akram@ies.univ-montp2.fr; 5 The State Key Laboratory of Power Transmission Equipment and System Security and New Technology, Chongqing University, Chongqing 400044, China; shoaibbhutta@hotmail.com; 6 School of Materials Science and Engineering, Jiangsu University, Zhenjiang 212013, China; shahid@ujs.edu.cn; 7 Institute for Tropical Technology, Vietnam Academy of Science and Technology, VAST, 18 Hoang Quoc Viet, Cau Giay, Hanoi 122300, Vietnam; ntanh@itt.vast.vn

**Keywords:** nanocomposites, dry band arcing (DBA), finite element analysis (FEA), carbon track, tracking lifetime, thermal performance

## Abstract

Ethylene propylene diene monomer (EPDM) is broadly employed as an insulating material for high voltage applications. Surface discharge-induced thermal depolymerization and carbon tracking adversely affect its performance. This work reports the electrical field modeling, carbon tracking lifetime, infrared thermal distribution, and leakage current development on EPDM-based insulation with the addition of nano-BN (boron nitride) contents. Melt mixing and compression molding techniques were used for the fabrication of nanocomposites. An electrical tracking resistance test was carried out as per IEC-60587. Simulation results show that contamination significantly distorted the electrical field distribution and induced dry band arcing. Experimental results indicate that electric field stress was noticed significantly higher at the intersection of insulation and edges of the area of contamination. Moreover, the field substantially intensified with the increasing voltage levels. Experimental results show improved carbonized tracking lifetime with the addition of nano-BN contents. Furthermore, surface temperature was reduced in the critical contamination flow path. The third harmonic component in the leakage current declined with the increase of the nano-BN contents. It is concluded that addition of nano-BN imparts a better tracking failure time, and this is attributed to better thermal conductivity and thermal stability, as well as an improved shielding effect to electrical discharges on the surface of nanocomposite insulators.

## 1. Introduction

Polymeric insulators are emerging as a mature technology and outstanding insulating solution for power equipment that is engaged in outdoor electricity networks. An outstanding flashover characteristic is a key attribute for their preference over conventional non-polymeric technology [1,2]. Among polymeric materials, ethylene propylene diene monomer (EPDM) is a popular choice after silicone rubber for outdoor insulators because it provides a better flexible design and pollution performance. In addition, EPDM insulation-based cables are used in nuclear radiation environments due to their superior resistance to gamma irradiation [3]. Polymeric materials have been found to render much better performance in highly polluted and contaminated sites. However, electrical discharge-induced carbonized conductive tracking is yet to be completely resolved due to the thermal aspects of the materials that are used in these scenarios.

It is well known that polymeric materials are prone to deteriorate under contaminated environment conditions due to the presence of electrical discharges [4]. Considerable leakage current can occur on a polluted wet surface of insulators. This current produces ohmic heating and the formation of dry band arcing (DBA) [5,6]. Incessant DBA activity destroys the surface hydrophobicity, which is considered as the first barrier against moisture ingress. Moreover, this incessant activity substantially enhances the electric field across dry bands [7]. This activity builds up the thermal stress and significantly escalates the temperature on the DBA area. This may lead to the thermal depolymerization of polymeric materials [8]. A polymeric structure may resist degradation up to a certain temperature, but, beyond that limit, it may undergo chemical alterations such as deterioration in the form of carbonized tracking.

A number of studies have been carried out on the tracking and erosion problem of polymeric materials over the past thirty years [9,10,11]. El-Hag reported the erosion resistance of polydimethylsiloxane (PDMS)-based silicone rubber-filled microns and nano-sized silica additives in an inclined place test (IPT) and concluded that five times lesser amounts of nano additives perform as well as micron silica additive-filled PDMS [12]. Another study by Ramirez reported the performance of PDMS filled with micron/nano additives and a surfactant in salt fog, IP, and ablation tests, and it was found that the surfactant improved the overall performance of PDMS [13]. 

As far as the electrical performance of EPDM is concerned, a number of studies have been conducted. Cheol compared performance in relation to the size of alumina trihydrate (ATH) particles [14] and found the tracking performance of EPDM declined while dielectric and water resistance properties improved with increasing particle size. Kurata explored the tracking performance of EPDM filled with ATH and carbon black particles [15]. It was demonstrated that tracking was dependent on particle size. Moreover, carbon black showed potential to improve weathering resistance. Lee [16] optimized the level of additive contents in order to enhance the tracking resistance of EPDM. With the increasing contents of ATH, EPDM performed better, and carbon black with a 1.5 pph positively impacted its tracking performance. Additionally, Ramirez found that EPDM filled with ATH additives performed better relative to its silica-filled counterparts [17]. Prabu [18] compared the tracking resistance performance of an EPDM/PDMS blend with individual polymers. Rajini [19] reported improved tracking and arc resistance performance in an EPDM/PDMS blend by exposing it to e-beam irradiation. Most recently, Fairus [20] explored the tracking performance of an EPDM/PDMS blend that contained nano Al_2_O_3_ and TiO_2_ particles. It was reported that the blend with nano additives performed well in the IPT and the 3 vol % of TiO_2_ performed equivalent to the 1 vol % of Al_2_O_3_. Refat compared the performance of EPDM with PDMS in the IPT under DC stress, and more early failure was seen in the −DC than +DC tested samples, regardless of the polymer type [21].

As far as the thermal depolymerization of EPDM is concerned, the literature is sparse. Meyer [22] investigated the correlation among degradation, discharge energy, and heat gain in the IPT for PDMS-filled ATH additives. Additionally, it was reported that the third harmonic component of leakage current was the key element that caused temperature escalation on the surface of the PDMS. Another study suggested that better thermal conductivity imparts better performance to PDMS and suppresses the DBA process [23]. A recent work by the authors compared the performance of PDMS filled with ATH/aluminium nitride (AlN)/boron nitride (BN) composites in the IPT [24]. It was shown that BN imparted significant improvement in the tracking/erosion performance followed by ATH due to its better thermal conduction ability. On the other hand, AlN-filled PDMS composites performed poorly, just like pure PDMS.

To date, although studies have been done on the thermal depolymerization of PDMS-based silicone rubber, no work has been reported to describe the heat accumulation on EPDM-based insulation in the IPT. Therefore, the present simulation and experimental study investigates the tracking lifetime of EPDM/BN nanocomposites along with infrared thermal analysis, leakage current, and electric field modeling. In addition, the role of thermal stability and thermal conduction characteristics are described to support the results.

## 2. Materials and Methods 

### 2.1. Nanocomposites Fabrication

**Figure 1:**
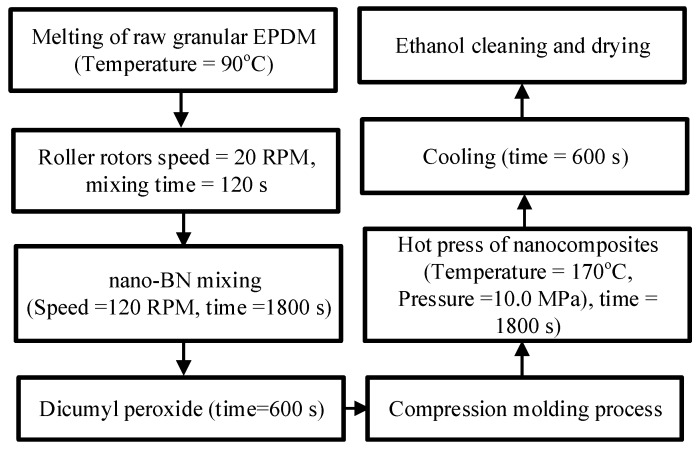
Step involved for the fabrication of the ethylene propylene diene monomer (EPDM) nanocomposite.

**Figure 2:**
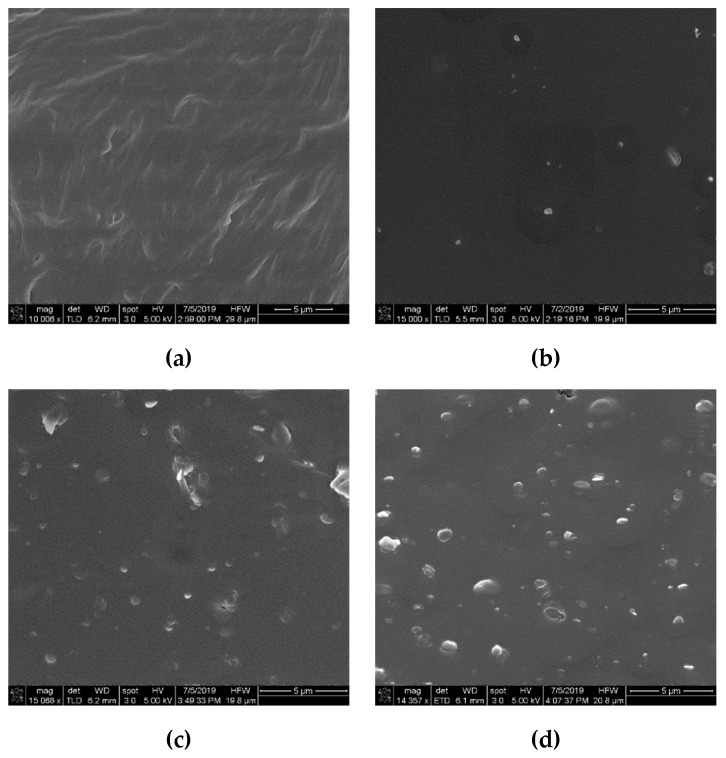
Nano-BN (boron nitride) distribution in (**a**) 0 wt %, (**b**) 1 wt %, (**c**) 5 wt % and (**d**) 7 wt % nanocomposites.

For nanocomposite synthesis, a pristine EPDM batch (Keltan^®^2470) with a density of 860 kg/m^3^ was used in this study. This pristine pellet form of EPDM was composed of ethylene (69%) and a bicyclic monomer ethylidene norbornene (4.2%). Boron nitride (BN) thermally-conductive nanoparticles [25] with a size of 50 nm and a density of 2250 kg/m^3^ were supplied by Beijing DK Nano Technology, Beijing, China. The surface of the nanoparticle was functionalized by KH570 silane coupling to reduce the degree of agglomeration in the composites. EPDM was crosslinked by adding dicumyl peroxide (DCP). The complete methodology of nanocomposites’ preparation is described in Figure 1. In order to determine the nano-BN dispersion in the composites, SEM micrographs of selected samples are shown in Figure 2. Nano-BN particles were seen to be homogeneously distributed in the samples, while some degree of agglomeration was observed in the nanocomposites with loadings of 5 and 7 wt % due to the attraction effect of adjacent nanoparticles under high contents. Finally, all the samples were cleaned with ethanol and rested overnight in an oven before the experiments.

### 2.2. Electrical Tracking Test

**Figure 3:**
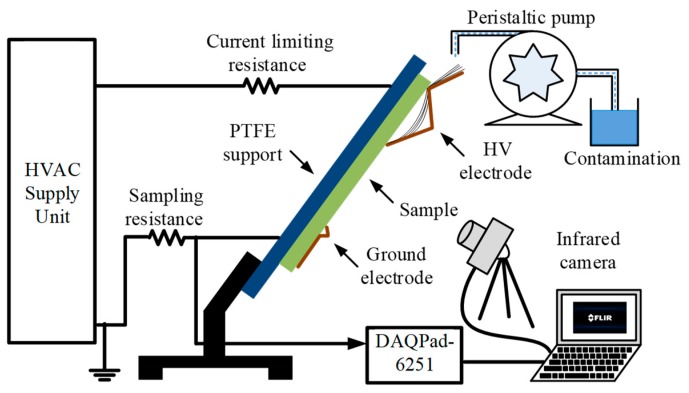
Inclined plane tracking test setup.

A protocol defined in IEC-60587 was used for the IPT [26], and a schematic diagram of the experimental arrangement is shown in Figure 3. The samples for the IPT were cast according to the standard with a size of 5 × 12 × 0.6 cm^3^, and five samples of each category were tested in order to estimate the failure criterion. The EPDM nanocomposites were mounted on an electrically-insulated polytetrafluoroethylene (PTFE) support. The top high voltage and bottom ground electrodes were mounted, and eight layers of filter papers were also inserted in the top high voltage electrodes to get a smooth contamination liquid path on the sample. The contamination liquid was prepared by mixing NH_4_Cl salt and Triton X-100 (Sigma-Aldrich, St. Louis, MO, USA) into deionized water. The electrical conductivity of the contaminant solution was adjusted at 2.50 mS/cm as per the standard. Protocol 2 of IEC-60587 was used with a starting applied voltage of 3000 V, and this was increased by 250 V per hour until the carbonized tracking failure criterion was achieved. The flow of contamination on the peristaltic pump was controlled at 0.30 mL per minute.

### 2.3. Tracking Life and Leakage Current

The tracking failure criterion A was used for the evaluation of the tracking lifetime, as per IEC Standard 60587. A timer device with an accuracy of ±1 min per hour was engaged to measure the failure time. Moreover, the leakage current was considered a prime indicator in order to analyze the tracking failure behavior of the insulation. A sampling resistor of 0.10 kΩ was used to measure the voltage drop. A 32-bit DAQPad-6251 (National Instruments, Austin, TX, USA was coupled with a PC to record the signal. The signal was digitized at a 10 kS per second sampling rate, and a MATLAB code was used to calculate the leakage current and its third harmonic component.

### 2.4. Thermal Distribution Studies

Thermography was carried out by using an infrared camera (FLIR E60, Wilsonville, OR, USA) during the IPT. The FLIR ResearchIR software was used for the post thermal analysis of the thermographs. Thermal distribution profiles were analyzed on a horizontal line that passed through the hot spot on the thermographs according to methodology defined in reference [27].

### 2.5. Thermal Stability and Thermal Conductivity

A TGA/SDTA 851 (Mettler Toledo, Zurich, Switzerland) was used to compare the thermal stability of the nanocomposites with thermogravimetric analyzer (TGA) and derivative thermogravimetric (DTG) profiles. The weight of each sample was taken as 8.0–12.0 mg. The temperature was linearly increased at a rate of 30 °C/min from 100 to 700 °C. Furthermore, the thermal conductivity of the nanocomposites was measured by using an LFA447 (Netzsch, Selb, Germany). The sample size for thermal conductivity measurement was controlled at 1 × 1 × 0.1 cm^3^.

## 3. Results and Discussion

### 3.1. Electric Field Simulation

**Figure 4:**
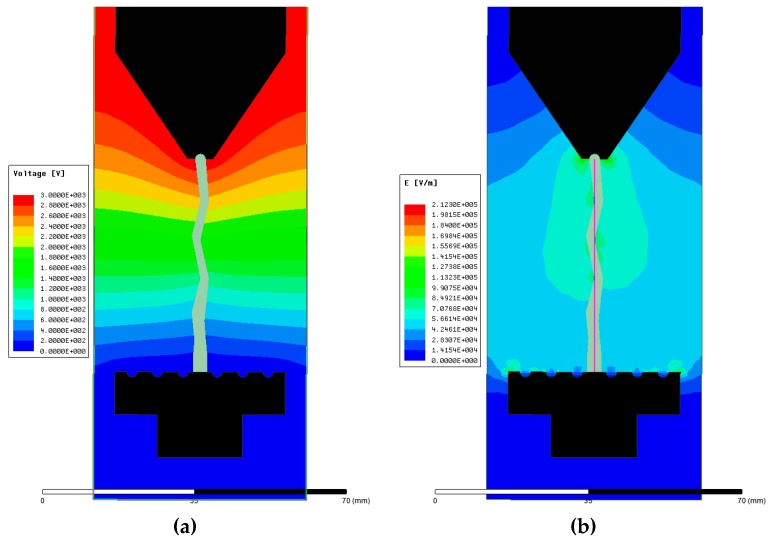
Voltage (**a**) and electric field distribution (**b**) in the inclined place test (IPT).

**Figure 5:**
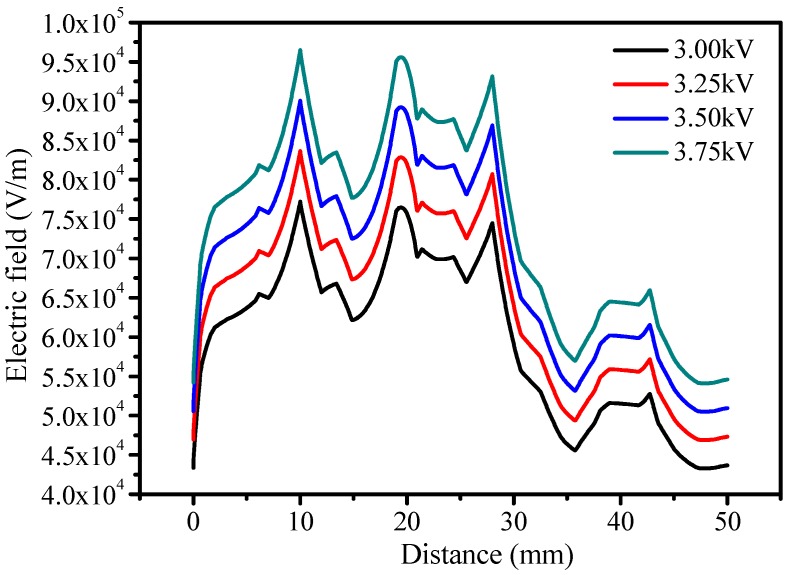
Electric field profiles at different voltage levels.

The ANSYS Maxwell^®^ finite element analysis (FEA) software (2017.1, ANSYS, Inc., Pittsburgh, PA, USA) was used for the electric field and voltage distribution modeling of the EPDM insulation. IEC 60587 was used as a reference for the modeling of the parameters in the simulation (Table 1). A 2D EPDM insulation model with the size of 5 × 12 cm^2^ was designed along with steel high voltage and ground electrodes as per the standard, as shown in Figure 4. Field modeling was performed and validated at the complete formation of the contamination path according to IEC Standard 60587. The electrical conductivity and relative permittivity of the modelled items were required in the simulation. The relative permittivity of EPDM was measured through a dielectric spectroscopy experiment. The electrostatic solution type was validated in the 2D analysis. Figure 4a shows the voltage distribution at the complete development of the contaminated path. The applied voltage gradually reduced from the top to the bottom electrode. The electric field distribution on the surface of the sample is shown in Figure 4b. It can be noticed that electric field stress was elevated at the edges of the contamination. Figure 5 shows the profiles of the electric field distribution on a vertical line bisecting the sample (shown in Figure 4b) from the top to the bottom electrode. The results show that electric field strength was substantially higher at the intersection of the sample surface and the tapered section of the contamination.

### 3.2. Carbon Tracking

**Figure 6:**
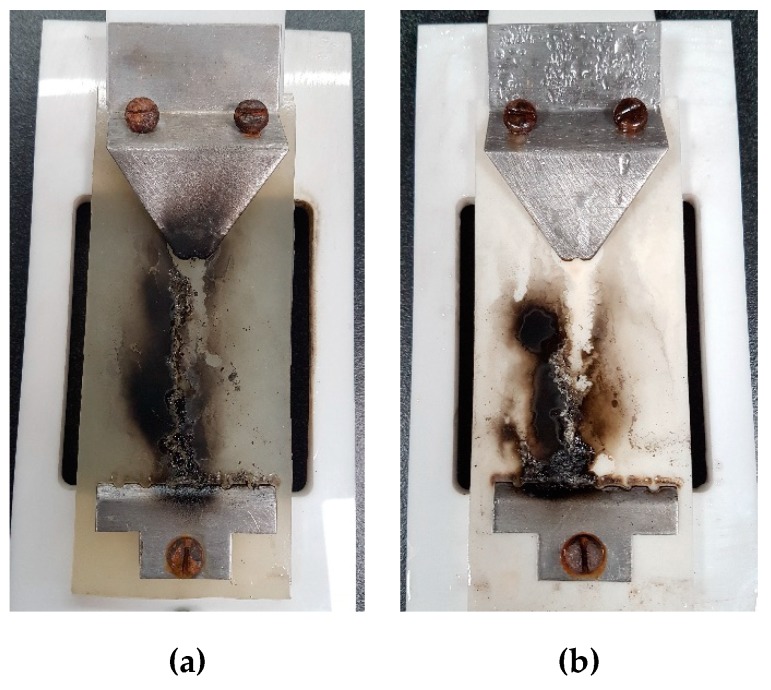
Carbonized tracking failure of the (**a**) 0 wt % and (**b**) 5 wt % samples.

**Figure 7:**
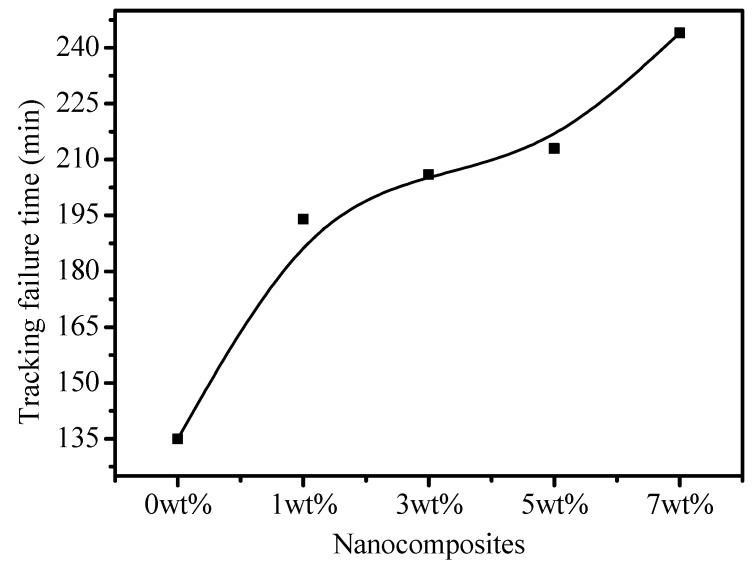
Carbonized tracking lifetime of the EPDM samples.

Carbon tracking arises from a thermally decomposed carbon contents due to the high amount of heat that is generated by electrical discharges. The release of high energy during the IPT process initiates the thermal depolymerization of EPDM, and carbon tracking tends to start from the bottom electrode and grow towards the high voltage upper electrode. Figure 6 shows the formation of the carbon track and the failure pattern on the surface of selected samples. It is clearly seen that a carbon path, which was electrically conductive, was formed on the surface between the electrodes. The heat accumulation in the discharge area broke the C–H chemical bond in the EPDM, and it resulted in conductive carbon product on the surface of the EPDM. For comparison, the carbon tracking failure times of all the samples are shown in Figure 7.

The tracking lifetime of the EPDM samples was measured at 135.0, 194.0, 206.0, 213.0 and 244.0 min for 0, 1, 3, 5 and 7 wt % loadings, respectively, as seen in Figure 7. Interestingly, the tracking life increased with BN loading in the EPDM. The results indicate that carbon tracking failure was substantially halted by the BN nanoparticles in the composites. The tracking lifetime was significantly higher in the nanocomposites with a higher contents of BN. Surprisingly, a small addition of nano-BN in the 1 wt % sample imparted significant resistance to the track formation and enhanced the failure time. The reason for the excellent tracking lifetime of the EPDM/BN could have been due to better heat distribution in the nanocomposites.

### 3.3. Thermal Distribution

**Figure 8:**
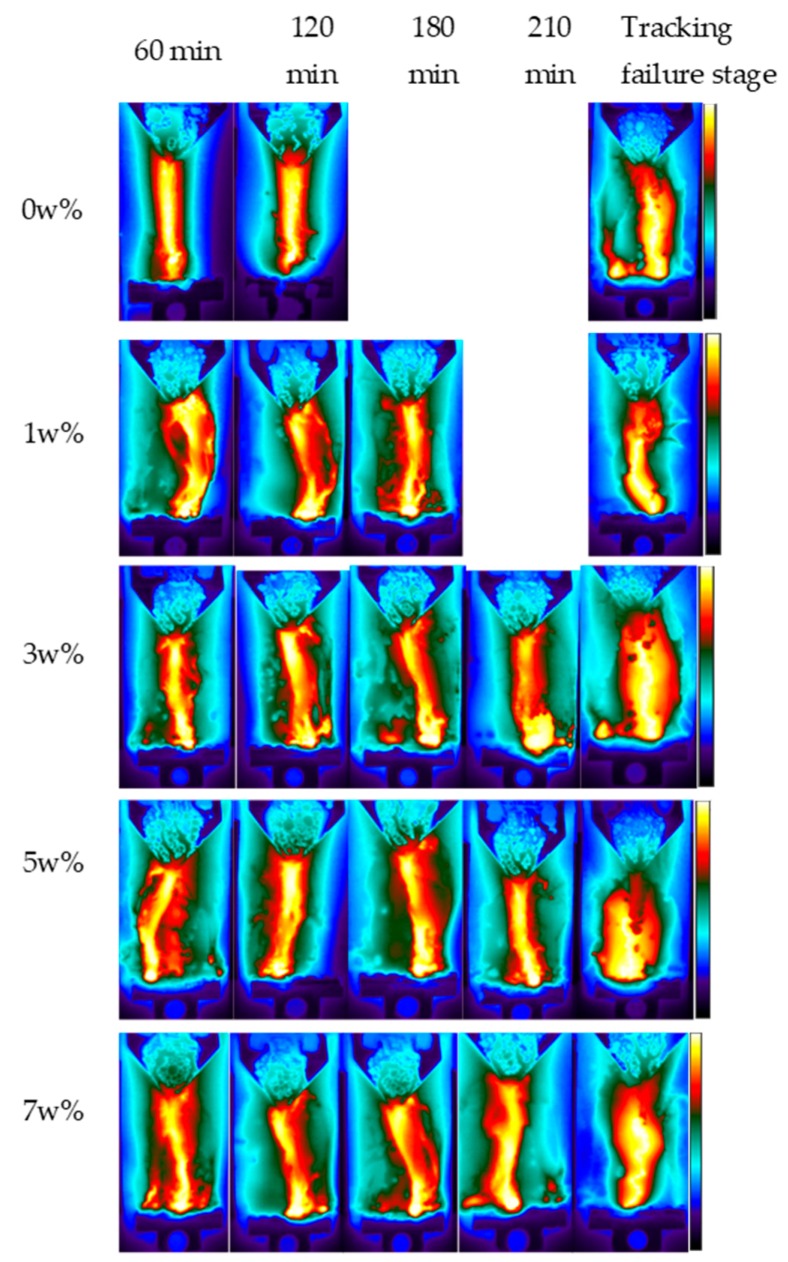
Infrared thermographs of the EPDM samples.

Carbon tracking failure occurs due to the thermal depolymerization of the insulating material due to excessive heat in the arcing area along the contamination path. Due to enhanced DBA activity with increasing voltage, the temperature on the surface of the sample varies on the journey of the tracking process. Here, infrared thermal micrographs were recorded over extended periods until the failure of each sample, as shown in Figure 8. Thermal accumulation activity mostly occurred in the central discharge area. Thermal accumulation was significantly enhanced with the IPT time lapse. The most severe DBA and thermal accumulation was exhibited at the failure stage of the samples.

**Figure 9:**
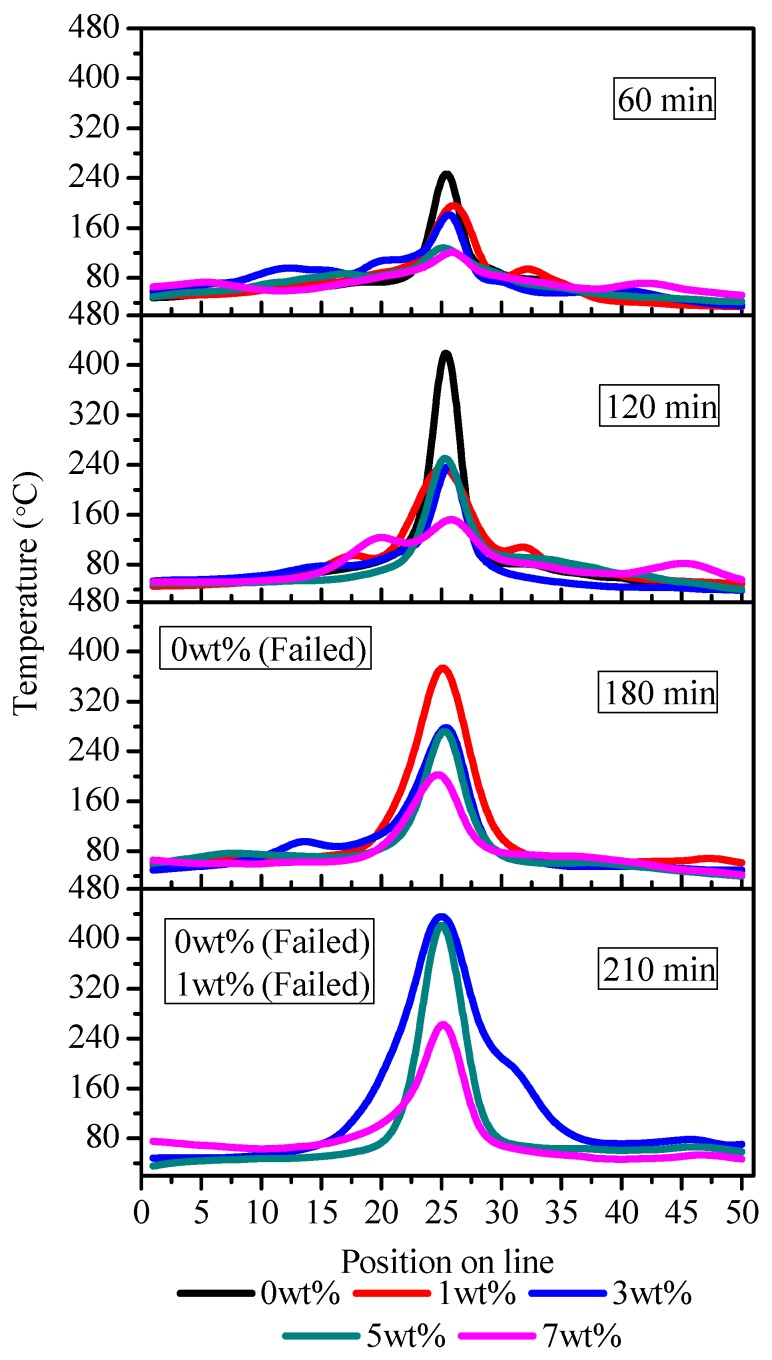
Thermal distribution profiles of the EPDM samples.

Figure 9 illustrates the thermal distribution of each sample according to the criteria described in Section 2.4. A higher thermal accumulation and a higher heat build-up in the central region are clearly witnessed in the infrared images. At the 60 min of the IPT, the recorded maximum temperatures for the nanocomposites were 256, 203, 190, 132, and 124 °C for the 0, 1, 3, 5 and 7 wt % samples, respectively. A significant increase on the surface temperature was seen at the end of 120 min of the tests for all samples except for that of 7 wt %. There was no failure seen in any sample at this point of the IPT.

A substantial thermal accumulation and a substantial temperature rise were seen in the third hour of the experiments. At 180 min, the temperatures were outstandingly increased to 386, 82, 283, 208 °C for the respective samples, as shown in Figure 9. The most interesting aspect of the results at this stage of the IPT was seen in a 0 wt %-unfilled sample, and it failed due to extreme DBA and thermal accumulation. At 210 min, the most severe DBA arcing and thermal accumulation activity was seen in the tests, and the temperatures were measured at 444, 446 and 275 °C for the 3, 5 and 7 wt % samples, respectively. Moreover, the 1 wt % sample also suffered carbonized tracking failure at this stage.

From experimental results, it can be conclusively said that temperature build-up was significantly reduced in the filled nanocomposites. It was noticed that the specimen could withstand up to a certain degree of temperature and thermal accumulation and then sharply tended to fail in the IPT. The critical temperature was 450 °C, and once the temperature exceeded this threshold, all samples showed little resistance to failure. It is interesting to notice that EPDM filled with nano-BN opposed thermal accumulation and resisted DBA activity on the surface; hence, it enhanced the tracking lifetime of the nanocomposites.

### 3.4. Leakage Current

**Figure 10:**
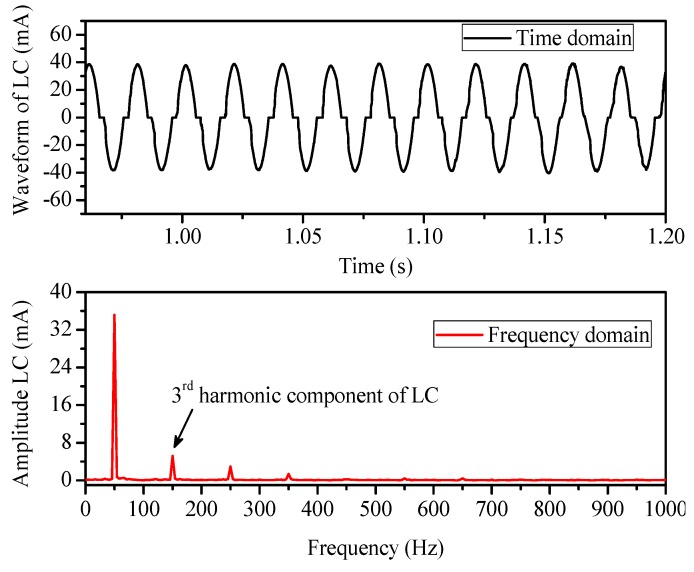
Time domain and frequency domain spectrum of leakage current.

The carbonized tracking failure and thermal accumulation in the IPT were correlated with the third harmonic component of the leakage current during DBA activity. Figure 10 shows the time domain and frequency domain amplitude of the leakage current during DBA. With the enhanced DBA activity, the third harmonic amplitude was increased significantly and caused major damage. Therefore, the third harmonic component of the leakage current was analyzed by using a fast Fourier transform in this study.

**Figure 11:**
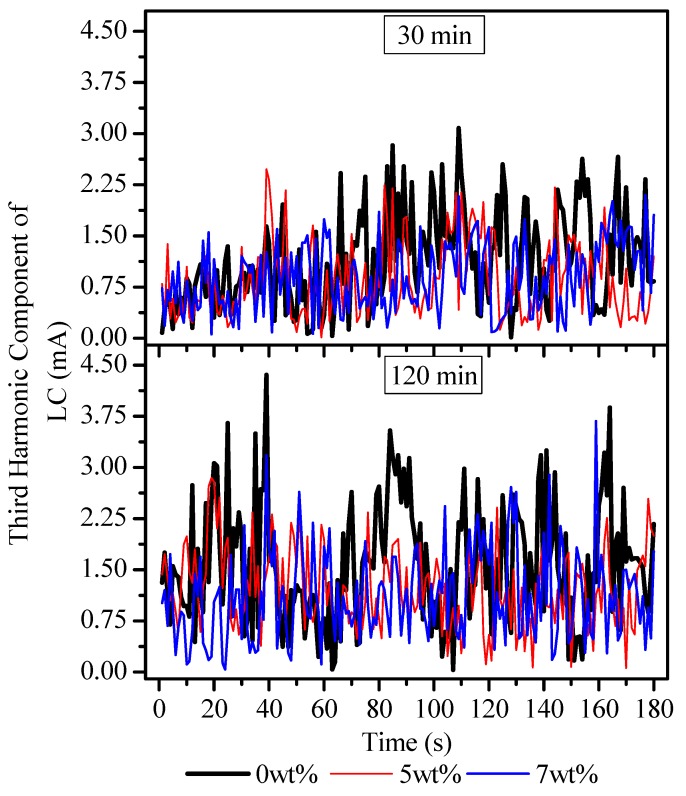
Third harmonic component of leakage current of the EPDM nanocomposites.

Figure 11 shows the third harmonic component of the leakage current of the samples. The result shows that the leakage current gained amplitude during the course of the IPT regardless of the nanoparticle loading. At 30 min of the IPT, the amplitudes were noticeably higher in the 0 wt % sample relative to its counterparts. Moreover, an increment in the peaks of leakage current were seen at 120 min of the IPT. As far as the particle loading is concerned, the amplitude of the third harmonic component in leakage current was seen to be consistent with leakage current results that were recorded at 30 min. Nonetheless, the 0 wt % sample attained a relative enhancement in the amplitudes of leakage current at 120 min, as it showed an increment in the DBA activity. This could have been due to the fast evaporation of the liquid contamination, which is a fundamental cause for this physical phenomenon. The results indicate that the EPDM filled with nano-BN could have restricted the third harmonic component in the leakage current, as compared to the unfilled 0 wt % EPDM. It is believed that nano-BN could have suppressed the third harmonic component in the leakage current on the surface of the nanocomposites and could have restricted the release of high energy. Hence, it impeded thermal depolymerization of the EPDM by suppressing the ohmic heating of the EPDM surface and prolonging the carbon track failure time.

### 3.5. Influence of Thermal Conductivity and Thermal Stability

**Figure 12:**
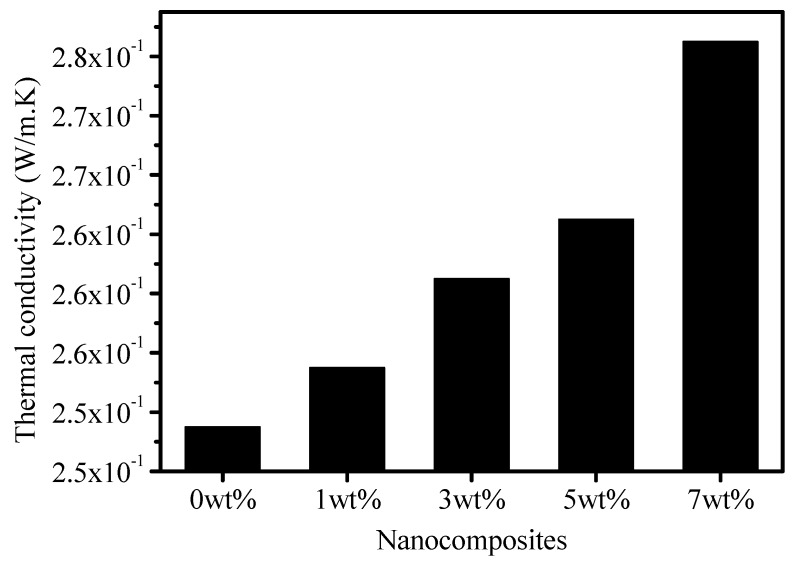
Experimental measured thermal conductivity of the EPDM nanocomposites.

A carbon track initiates due to a substantial amount of heat that is generated in discharge area, especially close to the bottom ground electrode, and it depolymerizes the chemical structure of EPDM. It is believed that long and severe DBA activity decomposes C–H bonds in EPDM, and this decomposition appears in the form of carbon, which produces an electrically conductive failure path on the surface [29]. A better thermal conduction in the discharge region could be a key player to suppress the thermal stress gradient in the discharge area [30]. Figure 12 shows the thermal conductivity of EPDM as a function of its nano-BN-filled counterparts. The results clearly suggest that the thermal conductivity of the filled counterparts was higher than that of the unfilled 0 wt % sample of EPDM. The thermal conductivity of the EPDM/BN nanocomposites increased with the addition of BN contents. The leakage current started to flow from the top to the bottom electrode, and, therefore, it was involved in the conduction, DBA, and heating of the surface. It is well known that a substantial degree of liquid evaporation occurs at the bottom electrode in an IPT, and it tends to enhance the dry band arcing in the region that initiates the carbonization. The EPDM/BN nanocomposites with higher thermal conductivity could conduct the heat out to the ambient from the discharge region and could suppress the temperature build-up. This process may have reduced the evaporation of liquid contamination on the surface of the nanocomposites. Therefore, it is highly likely that less evaporation on the surface of nanocomposites would reduce DBA activity and suppress discharge quantity [20]. On the other hand, less thermal conduction may enhance evaporation and increase DBA activity. Therefore, it is believed that nano-BN will assist in better heat conduction, less evaporation, reduced DBA, and better tracking lifetime of EPDM.

**Figure 13:**
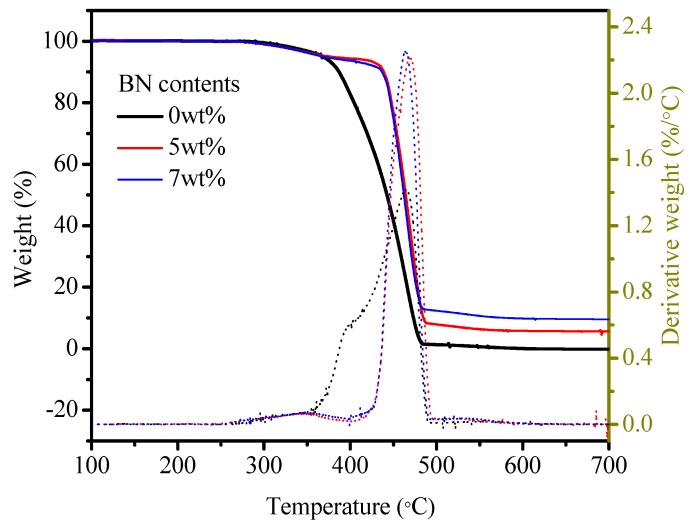
TGA; solid lines /DTG; dashed lines (derivative thermogravimetric) profiles of the EPDM nanocomposites.

The repetitive DBA process thermally depolymerizes EPDM; hence, the thermal stability of nanocomposites can play an important role in tracking performance [31]. Figure 13 shows the TGA/DTG profiles of the selected EPDM nanocomposites. The nanocomposites were found stable until 300 °C, and major degradation was observed beyond this temperature. The peak degradation was observed as the surface temperature exceeded 400 °C, which was consistent with infrared thermal analysis results. The initial degradation temperature (IDT) was measured as significantly higher in the filled nanocomposites relative to the 0 wt % sample, as shown from the DTG curves. Moreover, the primary thermal degradation temperatures of various weight losses of the nanocomposites are presented in Table 2. 

It is noted that the *T*_10%_, *T*_25%_ and *T*_50%_ weight losses of the filled nanocomposites were significantly higher than the unfilled 0 wt %. In addition, the percentage residual weight of the 0 wt % sample was almost zero at 700 °C, but it was significantly higher in the filled nanocomposites. From TGA/DTG results, it can be said that the carbonized tracking performance of the samples was directly correlated with the thermal stability performance of the nanocomposites. The nano-BN interaction with the EPDM matrix was the major factor that enhanced the thermal stability performance of the matrix. It is highly likely that a strong interaction between the EPDM matrix and nano-BN will enhance the chemical cross-linking and physical points, which will restrict segmental movement and improve thermal stability [24,32].

### 3.6. Influence of Interparticle Distance and Surface Area of Nano-BN

**Figure 14:**
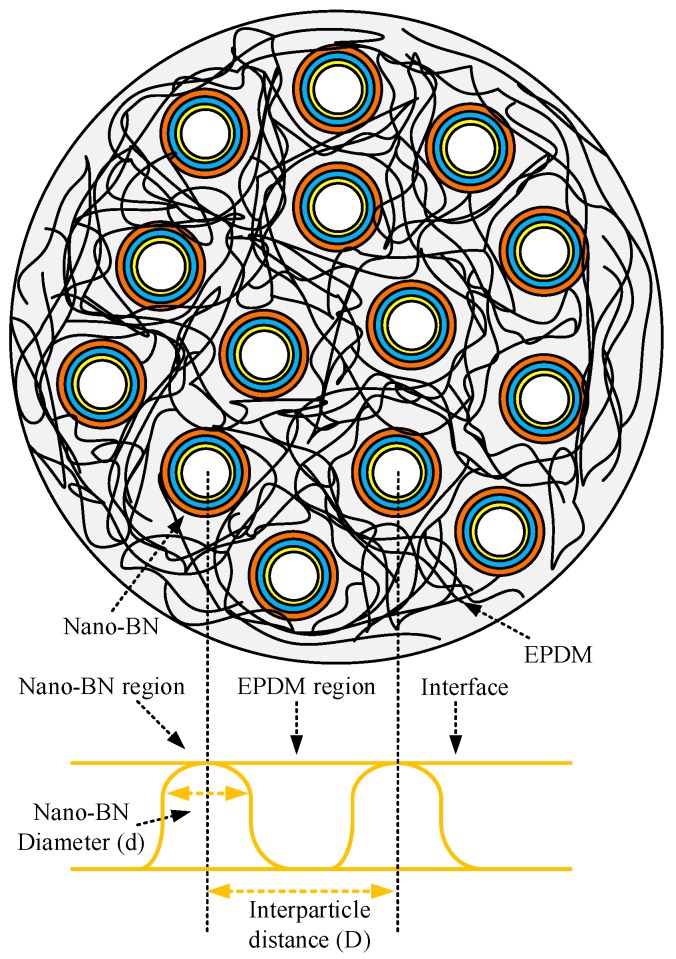
A model of nano-BN-filled EPDM.

Interparticle distances, the surface area of particles, and EPDM chain dynamics are key factors that play important roles in the tracking performance of EPDM. It is a common belief that interparticle distances sharply decline and the surface area of particles is drastically enhanced with increasing nanoparticle contents in composites. A proposed model of EPDM filled with spherical shape nano-BN is shown in Figure 14, and this illustrates the nano-BN region, the EPDM region, the interface, the particle size, and the interparticle distance. 

**Figure 15:**
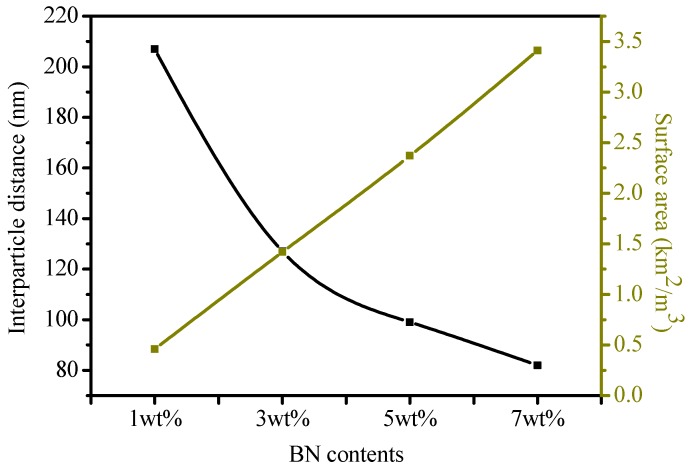
Results on mathematical calculation of interparticle distances and surface area of nano-BN.

Interparticle distances and the total surface area of the nano-BN particles in the nanocomposites were calculated according to the method provided in references [33,34] by assuming the ideal distribution, as shown in Figure 15. The interparticle distance between nano-BN declined whilst the total surface of nano-BN increased with the addition of BN contents. Realistically, there was a possibility of agglomeration in the nanocomposites, and interparticle distances can vary in the nanoscale range [8,35]. Moreover, it is expected that with increasing BN contents in nanocomposites, packing structure should be much better. Additionally, shorter interparticle distances will impart better shielding by reducing the chances of an EPDM matrix being exposed to dry band arcing.

## 4. Conclusions

The electrical discharge-induced carbonized tracking failure of nano-BN-filled EPDM was investigated with the inclined plane test. Electrical field simulation modeling, tracking lifetime, infrared thermal distribution, leakage current, thermal conductivity, and stability were studied in order to assess the performance of the EPDM nanocomposites. The following conclusions were extracted from the simulation and experimental results:

1. The electric field strength was substantially higher at the intersection of the sample surface and the edges of the area of contamination. An increasing field with increasing voltage intensified the dry band arcing activity, which initiated the ohmic heating-induced thermal depolymerization of EPDM and electrically conductive carbonized tracking.

2. The physical tracking lifetime of EPDM increased with the nano-BN contents. The higher the BN contents, the higher the tracking failure time. 

3. Infrared thermal analysis showed that temperature buildup in the discharge region was substantially reduced with the nano-BN addition. It is concluded that 450 °C was the critical surface temperature. Once the temperature exceeded this threshold, the samples showed little resistance to tracking failure. 

4. The third harmonic component of leakage current attained amplitude with the passage of the IPT, but the EPDM-filled nanocomposites performed comparatively better. 

5. The excellent carbonized tracking lifetime of the EPDM nanocomposites was attributed to improved thermal conductivity and thermal stability with nano-BN addition. The lower interparticle distances and higher total surface area of the nanocomposites resulted in better shielding against surface discharges.

## Figures and Tables

**Table 1:** Permittivity and conductivity parameters of materials.

Material	Relative Permittivity	Conductivity (S/m)
EPDM	2.43	2.02 × 10^−11^
Steel	1	11 × 10^5^
Contamination	81 [28]	0.020

**Table 2:** Thermal parameters of the nanocomposites extracted from TGA/DTG profiles.

Sample	% Weight Loss Temperature Points			% Residual Weight at 700 °C
	*T*_10%_	*T*_25%_	*T*_50%_	
0 wt %	387	412	442	0.00232
5 wt %	438	451	462	5.6
7 wt %	436	450	464	9.5
